# A Spanish adaptation of the Quality in Psychiatric Care—Inpatient (QPC-IP) instrument: Psychometric properties and factor structure

**DOI:** 10.1186/s12912-021-00710-3

**Published:** 2021-10-08

**Authors:** Sara Sanchez-Balcells, Maria-Teresa Lluch-Canut, Marta Domínguez del Campo, A. R. Moreno-Poyato, M. Tomás-Jiménez, Lars-Olov Lundqvist, Agneta Schröder, Montserrat Puig-Llobet, J. F. Roldan-Merino

**Affiliations:** 1 Community mental health nurse and case manager of the continuity of care program, Parc Sanitari Sant Joan de Déu, Sant Boi del Llobregat, Spain; 2 Department of Public Health, Mental Health and Maternal-Child Nursing, Nursing School, University of Barcelona, Health Sciences Campus Bellvitge, L’Hospitalet de Llobregat, Barcelona Spain; 3 Community mental health nurse, Parc Sanitari Sant Joan de Déu, Sant Boi del Llobregat, Spain; 4 Parc Sanitari Sant Joan de Déu, Sant Boi del Llobregat, Spain; 5 University Health Care Research Center, Faculty of Medicine and Health, Örebro University, Örebro, Sweden; 6 Department of Health Science, Faculty of Health, Care and Nursing, Norwegian University of Science and Technology (NTNU), Gjövik, Norway; 7 Campus Docent Sant Joan de Déu‑Fundació Privada, University of Barcelona, Barcelona, Spain

**Keywords:** Factor analysis, Inpatient psychiatric care, Nursing, Psychometric properties, Quality of care

## Abstract

**Background and aim:**

Western countries share an interest in evaluating and improving quality of care in the healthcare field. The aim was to develop and examine the psychometric properties and factor structure of the Spanish version of the Quality in Psychiatric Care–Inpatient (QPC-IP) instrument.

**Methods:**

A psychometric study was conducted, translating the QPC-IPS instrument into Spanish, revision of the instrument by a panel of experts, and assessing its psychometric properties. 150 psychiatric inpatients completed the QPC-IP. Test-retest reliability was assessed by re-administering the questionnaire to 75 of these patients.

**Results:**

After conducting pilot testing and a cognitive interview with 30 inpatients, it was determined that the QPC-IPS was adequate and could be self-administered. A Cronbach’s alpha of 0.94 was obtained for the full instrument and values of 0.52–0.89 for the various dimensions of the questionnaire. Test re test reliability: The Intraclass Correlation Coefficient for the full questionnaire was 0.69, while for the individual dimensions values between 0.62 and 0.74 were obtained, indicating acceptable temporal stability. Convergent validity was analysed using 10-point numerical satisfaction scale, giving a positive correlation (0.49). Confirmatory factor analysis revealed six factors consistent with the original scale. The Spanish version yielded adequate results in terms of validity and reliability.

**Conclusion:**

Our findings provide evidence of the convergent validity, reliability, temporal stability and construct validity of the Spanish QPC-IP for measuring patient quality in psychiatric care in Spanish hospitals. Hospital administrators can use this tool to assess and identify areas for improvement to enhance quality in psychiatric care.

## Background

Recent decades have seen a growing interest worldwide in measuring the quality of healthcare, not least in relation to mental health services [1]. However, these developments have not taken place to the same extent in all countries, nor even in different regions of the same country. One reasons for this is the lack of standardized measurement instruments for identify areas in need of improvement and for making national and international comparisons [2]. It has been observed in a recent systematic review [3] that quality instruments in mental health have psychometric properties with highly variable results and it is recommended to take into account those with high quality standards of results. For example, in the area of mental health the Quality of Psychiatric Care–Inpatient (QPC-IP) instrument [4] was developed with the aim of reduce this gap. The QPC-IP is part of a family of instruments that comprise both a common core and context specific: the QPC-OP for the psychiatric outpatient care [5], the QPC-FIP for psychiatric forensic inpatient care [6], the QPC-DA for daily activities in community-based services for people with psychiatric disabilities [7], and the QPC-H for the quality of community housing support [8]. Each QPC instrument also has a staff version, of which the one for psychiatric forensic inpatient care (QPC-FIPS) [9] has currently been validated. Adaptations of the QPC-FIP have been carried out in Denmark [10] and the QPC-IP and QPC-IPS in Indonesia [11, 12].

In Spain, legislation on the quality of public healthcare (Law 16/2003) has led to the creation of regional bodies with responsibility for assessing the quality of health services, especially mental health. In Catalonia, the Agency for Health Quality and Assessment (AQuAS) fulfils this role using validated satisfaction instruments. However, the concept of patient satisfaction does not encompass all aspects of quality of care. Questionnaires of satisfaction often involve questions that reflect the concerns of managers, such as health outcomes, rather than focusing on aspects which patients themselves might see as important [13]. Indeed, patient satisfaction is not necessarily synonymous with quality from the patient’s perspective [14], since measuring satisfaction in terms of service indicators is not the same as exploring patients definition of quality of care and what enable their recovery [15]. The essential components that make up this quality are the therapeutic setting, the therapeutic relationship and support, assessment, professional performance, assessment of practice, and environmental health [16].

Faced with this problem, the QPC-IP has the potential be a useful tool for assessing the quality of mental health services in Spanish-speaking countries. However, the QPC-IP was developed in the Swedish context, and according to the 6-D model of national culture [17], Spain and Sweden are culturally distinct, especially on the dimensions of uncertainty avoidance and masculinity-femininity, although they are similar in terms of power distance, individualism-collectivism, and long-term orientation. Given these cultural differences, a rigorous adaptation of the instrument is required, including analysis of its psychometric properties.

This research is part of an extensive study designed to adapt the instrument to other international contexts, analyze the psychometric properties and dimensions of the different instruments of the QPC-IP, and detail and compare the quality of hospital mental health care in the different territories.

## Methods

The adaptation process and the psychometric evaluation was carried out in the context of two psychiatric services in Catalonia, one in the city of Barcelona and the other in the nearby town of Sant Boi de Llobregat. Meetings were held with staff to inform them about the purpose and nature of the study. Nurses and occupational therapists recruited patients who were willing to participate and who met the inclusion criteria. After obtaining informed consent, the staff instructed the patients on how to complete the questionnaire. Patients were included consecutively until the required sample size was reached.

### Adaptation of Spanish inpatient instrument

**Fig. 1:**
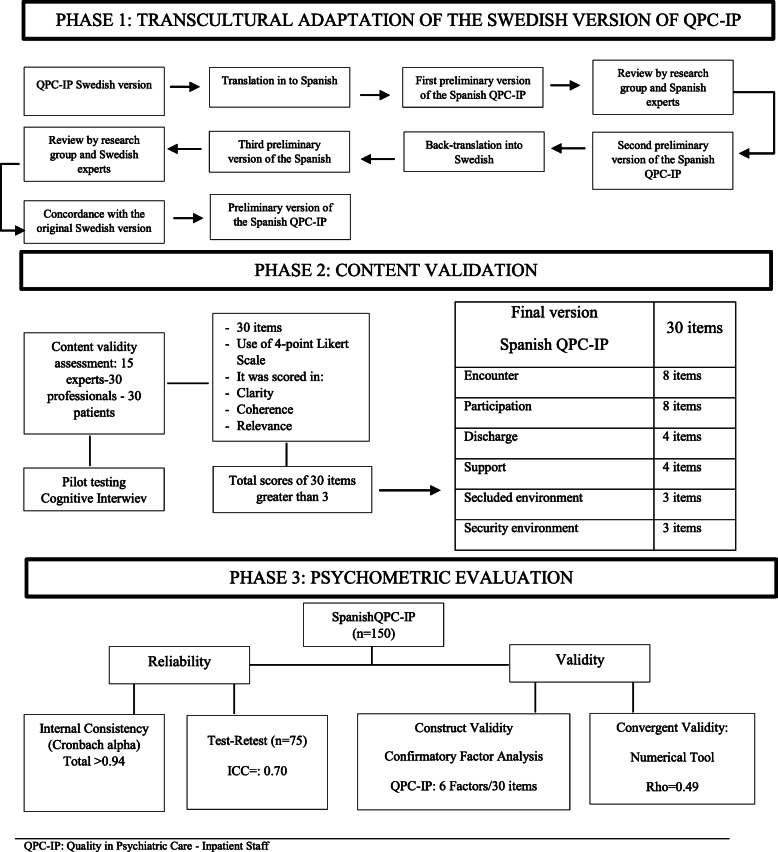
Overview of the three-phase validation study

Figure 1 shows the process of translation and back-translation of the development of the Spanish QPC-IP. The original (Swedish) version of the instrument was first translated into Spanish. The research team, comprising professionals from the fields of nursing and psychiatry, as well as health and care quality managers, reviewed the translation and checked that the meaning of each item was expressed and translated correctly (cultural validation). Each item was rated on a scale from 1 to 4 (minimum-maximum) with regard to its coherence, clarity, and relevance. This preliminary Spanish version was then back-translated into Swedish and sent to the Swedish research group. The Swedish research group assessed the degree of convergence between the back-translation and the original version. Following discussion and subsequent agreement regarding the semantic equivalence (face validity) of the Spanish QPC-IP the instrument was piloted in cognitive interviews with 30 psychiatric inpatients. This process confirmed that the Spanish version of the instrument was easy to understand and to answer.

### Analysis of psychometric properties

#### Sample and participants

The required sample size was estimated in accordance with the COnsensus-based Standards for the selection of health Measurement Instruments (COSMIN), the Standards for Educational and Psychological Testing and the judgment of experts [18]. It was considered that a minimum of 5 patients per questionnaire item would be needed to assess internal consistency. For the analysis of temporal stability, we estimated that a minimum of 75 patients would be required and that the intraclass correlation coefficient between the two administrations should be ≥.70 [19], considering a 95% confidence level and power of 80% in a two-sided test [20].

The total sample therefore comprised 150 inpatients from different units of the two psychiatric services (acute, sub-acute, long-stay, therapeutic community as an open door unit with a three-month average stay, residential care where patients live permanently). Data were collected between September and December 2017. The inclusion criteria were [1] being an inpatient in one of the aforementioned units at the time of the study, [2] aged 18 or over, [3] having a diagnosed mental disorder, and [4] voluntary participation. The exclusion criteria were [1] unable to understand or communicate in Spanish, [2] significant cognitive impairment, [3] currently in seclusion, [4] currently under mechanical and/or pharmacological restraint, [5] learning disability, [6] organic disorder and/or intoxication due to drug use.

#### Sample description

A total of 150 patients (45.3% female, 54.7% male) aged between 18 and 72 (mean = 43.6, SD = 12.63) completed the QPC-IP. The 86% of the sample were Spanish. Regarding marital status, 62% of admitted patients were single and 15.3% divorced or widowed. Regarding the education received, 26.6% completed primary school or did not finish them, and 30.7% of the population specialized in job training. In relation to the main occupation, 44% received the sickness pension. The average number of days of admission was 43 (SD 11.0). The 73.3% of the sample had been admitted in the hospital more than once. The average number of readmissions corresponded to 5.89 (SD 9.89). 65.3% of the total knew their diagnosis. According to the type of admission, 53.3% had a voluntary admission.

### The instruments

#### The quality in psychiatric care-inpatients

The QPC-IP was developed by Schröder et al. [21] for use in a specific mental health context and with the aim to give the patients’ views a central role in the assessment of psychiatric care quality. QPC-IP is a 30-item self-administered instrument based on a definition of quality of care from the patient’s perspective [3] that was developed through a phenomenographic interview study with inpatients and outpatients. The items cover six dimensions of psychiatric care, labeled as follows: Encounter (8 items), Participation (8 items), Support (4 items), Discharge (4 items), Secluded Environment (3 items), and Secure Environment (3 items). The Cronbach’s alpha values of the six dimensions ranged between .75 and .95. Each of the 30 items of the QPC-IP is rated on a 4-point Likert-type scale ranging from 1 (totally disagree, indicative of lowest quality) to 4 (totally agree, indicative of highest quality), and thus the total score ranges from 30 to 120. The definition was developed from a phenomenographic interview study [22], and the instrument was tested for face validity in a pilot study and also empirically tested [4].

#### 10-point numerical scale satisfaction

A numerical scale of satisfaction scores has been used, ranging from 0 to 10, with 0 being the lowest score and 10 the highest score being the best satisfaction score.

### Data analysis

The reliability (internal consistency) of the Spanish version of the QPC-IP was tested by computing Cronbach’s alpha coefficients for the total questionnaire and for each of its six dimensions. A value above .70 was considered adequate [23]. To test for scale homogeneity we calculated corrected item-total correlation coefficients, that is, the correlation of each item with the total score and with the score on each dimension when that item is omitted, accepting a correlation of .30 as the lower limit [23].

Test-retest reliability (temporal stability) was assessed over an interval of 7–14 days, re-administering the Spanish QPC-IP to 75 of the total of 150 patients. For this analysis we calculated the intraclass correlation coefficient (range 0 to 1) and interpreted a value ≥.70 as indicating good agreement [19], considering a 95% confidence level and power of 80% in a two-sided test [20].

To analyze convergent validity we calculated the Spearman (rho) correlation coefficient between scores on the Spanish QPC-IP and a 10-point numerical satisfaction scale on which 0 and 10 corresponded to the lowest and highest satisfaction rating.

Construct validity was tested by means of confirmatory factor analysis (CFA), with parameter estimates being obtained using the generalized least squares method and the EQS. 6.1 software package. This method is identical to the maximum likelihood estimator, but it has less strict normality criteria and is mainly used with ordinal items. The overall fit of the model was determined by calculating both absolute and incremental fit following indices: the GFI (goodness-of-fit index), the AGFI (adjusted goodness-of-fit index), the RMSEA (Root mean square error of approximation), the TLI (Tucker-Lewis index for comparison), the CFI (Comparative fit index), the BBNFI (Bentler-Bonett normed fit index), and the BBNNFI (Bentler-Bonett non-normed fit index). The criteria for a good fit were values of the GFI, AGFI, TLI, CFI, BBNFI, BBNNFI >.90, and a value of the RMSEA <.08 [24, 25]. We also calculated the reduced chi-squared statistic, defined as the ratio of the chi-squared value to the number of degrees of freedom. Values between 2 and 6 were considered acceptable [26].

A 95% confidence level was used for all the aforementioned statistical tests. Descriptive statistics were analyzed using SPSS 22.

## Ethical considerations

The study was approved by the Clinical Research Ethics Committee of our hospital (CEI PIC− 128 − 15) and permission was granted by the coordinators and supervisors of the respective psychiatric units. All questionnaires were confidential, and all the patients signed informed consent in accordance with existing Spanish legislation. Their participation was voluntary.

## Results

### Adaptation of Spanish inpatient instrument

For the adaptation of the Spanish instrument, a panel of experts produced the results based on coherence, clarity, and relevance greater than 3. No items required modification. After conducting a pilot testing and a cognitive interview with 30 inpatients, it was determined that the QPC-IPS was adequate and could be self-administered. The results of this phase were positive, and there were no problems in the comprehension or administration of the questionnaire.

### Analysis of psychometric properties

Table 1 shows the descriptive statistics of items from SpanishQPC-IP questionnaire.

**Table 1:** Descriptive Statistics of items from SpanishQPC-IP questionnaire

Items		Mean	SD	Medium	Kurtosis	Asymmetry	% Minimum Response	% Maximum Response
P1	I could influence my own care and treatment	2,93	0,92	3	−0,33	−0,64	10,0	30,0
P2	There was a high level of security at the ward	3,20	0,90	3	0,48	-1,10	8,0	44,7
P3	I had access to a place that was private where I could withdraw when I wanted to be left in peace and quiet	2,65	1,11	3	-1,24	−0,29	23,3	28,0
P4	I was secure together with my fellow patients	2,87	0,96	3	−0,54	− 0,60	12,7	28,7
P5	My opinion about what was the correct care and treatment for me was respected	3,04	0,91	3	0,04	−0,84	9,3	34
P6	I was involved in deciding about my care	3,05	0,93	3	-0,12	-0,82	9,3	36,7
P7	I received support and I had the opportunity to talk when I needed to	3,28	0,81	3	1,10	−1,18	5,3	45,3
P8	Hospital and community services co-operated when planning my future care and activities	2,92	0,95	3	−0,48	−0,63	11,3	31,3
P9	I was not disturbed by the other patients	2,60	1,03	3	−1,13	−0,08	16,7	24,7
P10	The staff were involved and were out there among the patients in the ward	3,20	0,85	3	0,45	−0,99	6	42
P11	The staff treated me with warmth and consideration	3,28	0,84	3,28	0,94	−1,20	6	47,3
P12	If I was angry and irritated the staff were concerned enough to want to know why	3,22	0,88	3,22	0,84	−1,19	8	43,3
P13	My previous experiences of medical treatment was utilised in the best possible way	3,06	0,85	3	0,74	−1,01	8,7	30,7
P14	I got to recognise signs of deterioration in my mental health	3,34	0,86	4	1,26	−1,39	6,7	53,3
P15	The staff respected me	3,51	0,70	4	2,81	−1,65	2,7	59,3
P16	I was offered a follow-up after discharge	3,29	0,88	3,29	1,29	−1,40	8	46
P17	Before I was discharged I received help to find an occupation	2,75	0,95	2,76	−0,44	−0,55	16	22,7
P18	The staff showed that they understood my feelings	3,02	0,93	3	−0,12	−0,81	10	34,7
P19	The staff prevented me from hurting those around me if I had such thoughts	3,11	0,79	3,11	1,43	−1,16	7,3	28,7
P20	The staff had the time to listen to me	3,28	0,80	3,28	0,98	−1,14	4,7	44,7
P21	I received information about where I could go if I needed help following discharge	3,18	0,91	3,19	0,75	− 1,21	10	41,3
P22	The staff prevented me from harming myself if I had such thoughts	3,21	0,83	3,21	1,33	−1,26	7,3	37,3
P23	The staff helped me to understand that it is not shameful to suffer from mental health problems	3,27	0,85	3,28	1,33	−1,33	7,3	44,7
P24	The staff helped me to understand that feelings of guilt and shame must never prevent me from seeking care	3,14	0,94	3,14	0,41	−1,11	11,3	40,7
P25	The staff were concerned about my care and treatment	3,33	0,81	3,33	1,94	−1,44	6,7	48
P26	There was the opportunity to have my own room	2,58	1,13	2,58	−1,30	−0,21	26,7	26,7
P27	I was informed in an understandable way about my mental health problems/diagnosis	2,84	1,06	3	−0,93	− 0,54	17,3	33,3
P28	There was a private place where I could receive visits from my next of kin	2,67	1,09	3	−1,20	−0,28	21,3	28,7
P29	I received information about my mental health problems in such a way that I could take part in my care	2,73	1,00	3	−0,91	−0,34	15,3	26,7
P30	I received information about different treatment alternatives so that I could decide which was best for me	2,69	1,06	3	−1,11	−0,29	18,7	28

*DS* Standard Deviation

### Construct validity

**Fig. 2:**
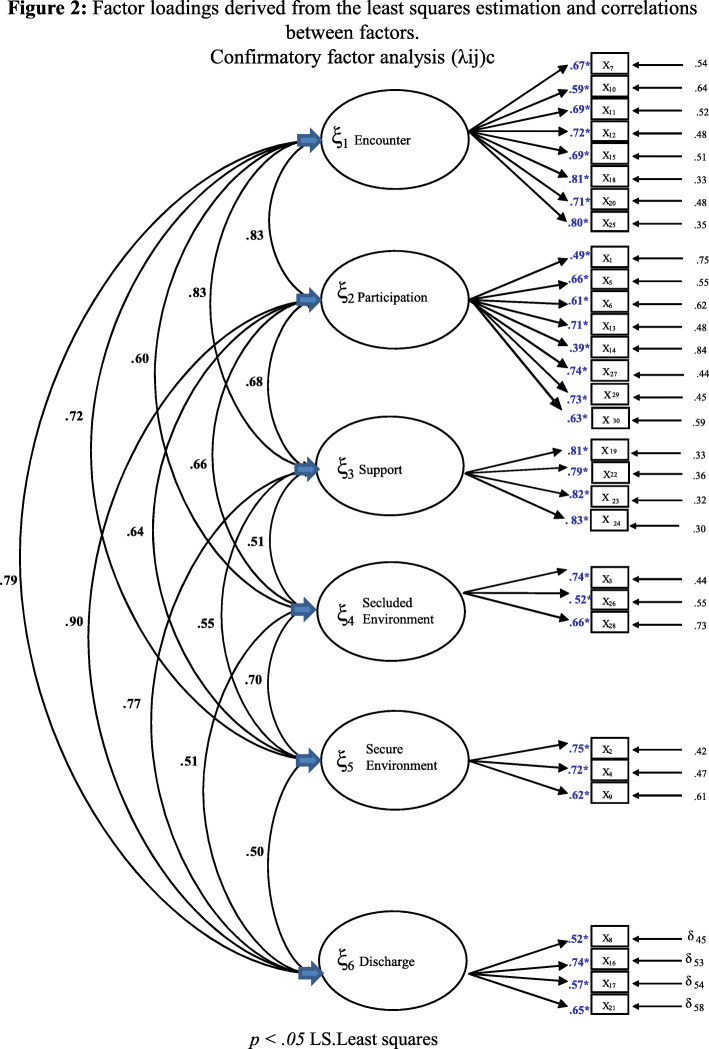
Factor loadings derived from the LS estimation (least squares) Confirmatory Factor Analysis (λij)

In the confirmatory factor analysis (CFA) all factors loadings were statistically significant (Fig. 2). The highest loadings were found for the Participation (Factor 1) and the Discharge dimensions (Factor 6). The correlations between factors were high in all cases, with the exception of that between Secure environment (Factor 5) and Discharge (Factor 6) (value of .50).

The result of the chi-square test was significant (χ^2^ = 935.500; *p* < .001), suggesting inadequate model fit. However, since the chi square test is sensitive to sample size we proceeded to calculate the ratio between chi-square and the degrees of freedom (χ^2^ / df). The value obtained was 2.39, wish is well within the 2 and 6 range considered to indicate acceptable fit [27]. As the results of our initial CFA indicated a positive latent variable covariance matrix. The results obtained ranged from adequate to excellent. Furthermore, the other fit indices confirmed an adequate fit of the model (Table 2).

**Table 2:** Goodness of fit indices of Model Confirmatory

INDEX	VALUE
BBNFI	.869
BBNNFI	1.001
GFI	.967
AGFI	.961
CFI	1.000
TLI	0.96
RMSEA	.000
Adjusted goodness test	χ2 = 935.500; df = 390; *p* < .0001
Adjustment reason	χ2 / df = 2.39

*BBNFI* Bentler Bonnet Normed Fit Index, *BBNNFI* Bentler Bonnet Non Normed Fit Index, *GFI* Goodness of Fit Index, *AGFI* Adjusted Goodness of Fit Index, *TLI* Tucker-Lewis index for comparison, *CFI* Comparative Fit Index, *RMSEA* Root mean square error of approximation, *Df* degrees of freedom

### Convergent validity

Analysis of the correlation between scores on the Spanish QPC-IP and a 10-point Numerical Scale Satisfaction yielded a Spearman’s rho coefficient of .49. The correlation of total and each subdimension of the QPC-IP was 0.86 (F1: Encounter), 0.89 (F2: Participation), 0.74 (F3: Support), 0.67 (F4: Seclude Environment), 0.63 (F5: Secure Environment), and 0.76 (F6: Discharge).

### Internal consistency

As shown in Table 3, the Cronbach’s alpha for the total scale was .94, and five of the six dimensions yielded a value above .70. The exception was ‘Secluded environment’, which received an alpha of .67. We also calculated alpha values for the total scale and each of its dimensions when excluding one item at a time. Internal consistency was not notably improved by excluding any of the items.

**Table 3:** Coefficient of internal consistency of SPANISH-QPCIP

Content of the summarized items		Alfa de Cronbach			Mean	SD
		Total subscale	Total subscale without item	Total scale without item		
Encounter		.891			3.26	0.62
7	I received support and I had the opportunity to talk when I needed to		.880	.940	3.28	0.81
10	The staff were involved and were out there among the patients in the ward		.890	.940	3.20	0.85
11	The staff treated me with warmth and consideration		.878	.940	3.28	0.84
12	If I was angry and irritated the staff were concerned enough to want to know why		.879	.940	3.22	0.88
15	The staff respected me		.876	.940	3.51	0.70
18	The staff showed that they understood my feelings		.868	.939	3.02	0.93
20	The staff had the time to listen to me		.878	.940	3.28	0.80
25	The staff were concerned about my care and treatment		.872	.939	3.33	0.81
Participation		.836			2.96	0.65
1	I could influence my own care and treatment		.826	.942	2.93	0.92
5	My opinion about what was the correct care and treatment for me was respected		.814	.940	3.04	0.91
6	I was involved in deciding about my care		.806	.940	3.05	0.93
13	My previous experiences of medical treatment was utilised in the best possible way		.813	.940	3.06	0.85
14	I got to recognise signs of deterioration in my mental health		.851	.942	3.34	0.86
27	I was informed in an understandable way about my mental health problems/diagnosis		.799	.939	2.84	1.06
29	I received information about my mental health problems in such a way that I could take part in my care		.805	.939	2.73	1.00
30	I received information about different treatment alternatives so that I could decide which was best for me		.814	.940	2.69	1.06
Support		.889			3.18	0.74
19	The staff prevented me from hurting those around me if I had such thoughts		.882	.940	3.11	0.79
22	The staff prevented me from harming myself if I had such thoughts		.857	.940	3.21	0.83
23	The staff helped me to understand that it is not shameful to suffer from mental health problems		.839	.940	3.27	0.85
24	The staff helped me to understand that feelings of guilt and shame must never prevent me from seeking care		.848	.939	3.14	0.94
Secluded environment		.679			2.64	0.87
3	I had access to a place that was private where I could withdraw when I wanted to be left in peace and quiet		.574	.941	2.65	1.11
26	There was the opportunity to have my own room		.652	.943	2.58	1.13
28	There was a private place where I could receive visits from my next of kin		.524	.942	2.67	1.09
Secure Environment		.739			2.89	0.79
2	There was a high level of security at the ward		.696	.941	3.20	0.90
4	I was secure together with my fellow patients		.509	.941	2.87	0.96
9	I was not disturbed by the other patients		.739	.942	2.60	1.03
Discharge		.712			3.04	0.68
8	Hospital and community services co-operated when planning my future care and activities		.702	.942	2.92	0.95
16	I was offered a follow-up after discharge		.610	.940	3.29	0.88
17	Before I was discharged. I received help to find an occupation		.693	.941	2.75	0.95
21	I received information about where I could go if I needed help following discharge		.586	.941	3.18	0.91
Overall Questionnaire		.942			3.05	0.56

### Test-retest reliability

Temporal stability was assessed by 75 drawn from the total of 150 patients. The intraclass correlation coefficient for the total scale was .69 (95% CI: .52–.81), ranging between .62 and .74 for the six dimensions (Table 4).

**Table 4:** Intraclass Correlation Coefficient (ICC) test-retest

Factors or dimensions of the questionnaire	ICC	CI 95%
F1: Encounter	0.869	0.802–0.913
F2: Participation	0.892	0.837–0.929
F3: Support	0.727	0.588–0.820
F4: Seclude Environment	0.853	0.778–0.903
F5: Secure Environment	0.856	0.782–0.905
F6: Discharge	0.681	0.518–0.789
TOTAL	0.911	0.865–0.941

*ICC* Intraclass Correlation Coefficient

*CI* Confidence Interval

### Descriptions of quality of inpatient care

**Fig. 3:**
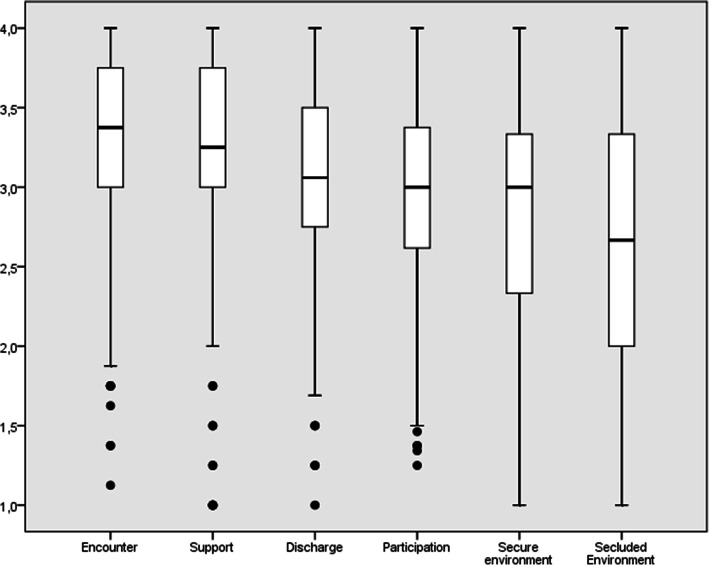
Mean ratings of the Spanish version of the QPC-IP dimensions. Error bars represent 95% Confidence interval

As shown in Fig. 3, the perception of quality based on the *Encounter* dimension was significantly higher than the second factor, *Support* (t_(149)_ = 3.18, *p* = 0.04), which was perceived as higher than *Discharge* (t_(149)_ = 3.04, *p* = 0.00). The remaining three dimensions were rated from highest to lowest; the *Participation* being greater than *Secure Environment* (t_(149)_ = 2.89, *p* = 0.23), and greater than *Secluded Environment* (t_(149)_ = 2.63, *p* = 0.00).

## Discussion

The purpose of this study was to develop and examine the psychometric properties and factor structure of a Spanish adaptation of the QPC-IP instrument. The results show that the Spanish QPC-IP has adequate psychometric properties in terms of internal consistency, temporal stability (test-retest), content and construct validity (confirmatory factor analysis) and convergent validity. The CFA revealed an adequate fit of the six-factor structure consistent with the original Swedish version.

It was determined that the instrument was adequate and could be self-administered after conducting a pilot test and a cognitive interview with 30 patients. The results of this phase were positive and there were no problems in the comprehension or administration of the questionnaire. Although 13.3% of the sample did not complete comprehensive school, no support was needed for the patients to complete the instrument.

Internal consistency was acceptable (α ≥ .70) for the total scale and for five of the six dimensions. The highest alpha value was found for the Encounter dimension and the lowest for Secluded environment dimension (α = .67). Since Cronbach alpha is highly influence by the number of items in a dimension, the low alpha value of Secluded environment is probably due to the fact that it only include three items. However, the internal consistency of Secluded environment in other language versions of the QPC-IP, such as the Indonesian one [12], also shows a low Cronbach alpha value, suggesting that this dimension might be more cultural specific than the other dimensions in the QPC-IP. Overall, the magnitude of the alpha values for individual items showed a reasonable degree of variation, suggesting that none of the items was perceived as confusing or upsetting. It should be noted that the alpha values are adequate according to established criteria [23] and are higher than those reported for the original version [21] and in other studies that have used the OPC-IP [11]. It is also worth pointing out that the reliability results obtained for the Spanish QPC-IP are similar to other quality of care measures reported in a recent systematic review [3]. Specifically, instruments yielding similar results to those of the Spanish QPC-IP are the SEQUenCE (Service user Quality of CarE) instrument [28], with a Pearson correlation coefficient of .65 and a Cronbach’s alpha of .87, the MQOC (Menninger Quality of Care) measure [29], with an alpha of .92, and the QPC-IPS with an alpha 0.92 [30]*.*

Regarding test-retest reliability, the value of the intraclass correlation coefficient obtained in the present study (ICC = .69) was acceptable. The Discharge dimension (ICC = .681) showed the lowest ICC values. One of the reasons for the low ICC values may be that patients discharge from ward were not scheduled in advance.

The convergent validity of the Spanish QPC-IP was examined by calculating the Spearman rho correlation coefficient with respect to a 10-point numerical satisfaction scale. The result was interpreted according to the criteria proposed by Martínez González, Sánchez Villegas, Toledo Atucha, and Faulin-Fajardo [31], namely: a value of zero, no correlation; <.30, weak association, ≥.31 ≤ .70, moderate association; >.71, strong association. The value obtained (rho = .49, *p* < .001) indicates a moderate association, showing that the higher the level of satisfaction the more positive is the patients’ rating of quality of care.

Regarding construct validity, the CFA yielded a six-factor model consistent with the original QPC-IP [21], thus confirming the multidimensional nature of the concept of quality [32]. All the item loadings were above .30, which is considered an acceptable minimum [33]. The goodness-of-fit indices also indicated a reasonably good fit of the model [25]. Overall, the results of the CFA support the validity of the Spanish QPC-IP and are in line with those reported for the QPC-IP [21], the QPC-OP [5] the QPC-FIPS [32] the Danish QPC-FIPS [34], and the Indonesian QPC-IP [12] and the Indonesian QPC-IPS [11].

It should be noted, however, that some differences were observed with respect to the recently validated Indonesian adaptation of the QPC-IP [12], possibly due to cultural differences with respect to our country.

However, given the present results, we can see that the patients in this study rated the highest quality in the *Encounter dimension*, which is in the line with previous studies on inpatient psychiatric care [12, 21]. The *Secluded Environment* was rated the lowest in terms of quality of care whereas in the study by Schröder et al. [21] performed in Sweden the *Secluded Environment* dimension was the second highest in terms of quality. One possible explanation may be that in Spanish psychiatric units there are no individual rooms available, except for isolation rooms for emergency crisis. However, it should be noted that the *Secluded environment* dimension in the Spanish QPC-IP fitted the original Swedish model whereas in the study by Lunqvist et al., [12], performed in Indonesia, the *Secluded Environment* dimension did not fit the model of the original scale. This result indicates that the concept of secluded environment is fairly similar in Spain and Sweden but distinctly different between Indonesia and Sweden, and possibly Spain too.

The extensive validation process in adapting the QPC-IP to the Spanish situation is a strength of the present study. However, a number of limitations also need to be acknowledged. First, although we estimated the minimum sample size needed for the present analysis, further studies with larger samples are required to confirm the factor structure obtained here. Second, the instrument has been developed with the specific purpose of assessing quality of care from the psychiatric inpatients’ perspective. Further studies are therefore needed to adapt the QPC instrument for use with Spanish-speaking populations in other contexts, such as outpatients. It would also be of interest to analyze the predictive capacity (sensitivity and specificity) of the Spanish QPC-IP instrument on patient recovery.

## Conclusion

The Spanish QPC-IP is a simple and easily administered tool for measuring various aspects of quality in psychiatric inpatient care from the patients’ perspective. The six-factor structure and psychometric property of the Spanish QPC-IP are consistent with those of the original instrument, supporting its use as a measure of quality of care in Spanish-speaking populations. In this respect, it has the potential be used in cross-cultural comparative studies of quality of care in mental health. The results of these studies can be used to improve the quality of the provided service. Future studies will need to look at the psychometric properties of this instrument in relation to other variables and other samples of patients, both in the community and in other settings.

## Data Availability

The datasets generated and/or analysed during the current study are not publicly available due to them containing information that could compromise research participant consent but are available from the corresponding author on reasonable request.

## Abbreviation

QPC-IP: Quality in Psychiatric Care-InPatients
