# Correction to: A Spanish adaptation of the Quality in Psychiatric Care—Inpatient (QPC-IP) instrument: Psychometric properties and factor structure

**DOI:** 10.1186/s12912-022-00829-x

**Published:** 2022-04-11

**Authors:** Sara Sanchez-Balcells, Maria-Teresa Lluch-Canut, Marta Domínguez del Campo, A. R. Moreno-Poyato, M. Tomás-Jiménez, Lars-Olov Lundqvist, Agneta Schröder, Montserrat Puig-Llobet, J. F. Roldan-Merino

**Affiliations:** 1 Community Mental Health Nurse and Case Manager of the Continuity of Care Program, Parc Sanitari Sant Joan de Déu, Sant Boi del Llobregat, Spain; 2 Department of Public Health, Mental Health and Maternal-Child Nursing, Nursing School, University of Barcelona, Health Sciences Campus Bellvitge, L’Hospitalet de Llobregat, Barcelona, Spain; 3 Community Mental Health Nurse, Parc Sanitari Sant Joan de Déu, Sant Boi del Llobregat, Spain; 4 Parc Sanitari Sant Joan de Déu, Sant Boi del Llobregat, Spain; 5 University Health Care Research Center, Faculty of Medicine and Health, Örebro University, Örebro, Sweden; 6 Department of Health Science, Faculty of Health, Care and Nursing, Norwegian University of Science and Technology (NTNU), Gjövik, Norway; 7 Campus Docent Sant Joan de Déu‑Fundació Privada, University of Barcelona, Barcelona, Spain

**Correction to: BMC Nursing 20, 191 (2021)**

**https://doi.org/10.1186/s12912-021-00710-3**

Following publication of the original article [1], the author noticed the author affiliations 2, 4 and 9 were the same department, they need to be unified to affiliation 2.

The correct author affiliations have been updated in this Correction.

The original article [1] has been updated.
